# The enigma of site of action of migraine preventives: no effect of metoprolol on trigeminal pain processing in patients and healthy controls

**DOI:** 10.1186/s10194-017-0827-x

**Published:** 2017-12-19

**Authors:** Julia M. Hebestreit, Arne May

**Affiliations:** Department of Systems Neuroscience, Center for Experimental Medicine, University Medical Center Eppendorf, Martinistr. 52, 20246 Hamburg, Germany

**Keywords:** Metoprolol, Beta-blocker, Thalamus, Migraine, fMRI, Pain, Pharmacological modulation, Preventive treatment, Nociceptive trigeminal system, Pain

## Abstract

**Background:**

Beta-blockers are a first choice migraine preventive medication. So far it is unknown how they exert their therapeutic effect in migraine. To this end we examined the neural effect of metoprolol on trigeminal pain processing in 19 migraine patients and 26 healthy controls. All participants underwent functional magnetic resonance imaging (fMRI) during trigeminal pain twice: Healthy subjects took part in a placebo-controlled, randomized and double-blind study, receiving a single dose of metoprolol and placebo. Patients were examined with a baseline scan before starting the preventive medication and 3 months later whilst treated with metoprolol.

**Results:**

Mean pain intensity ratings were not significantly altered under metoprolol. Functional imaging revealed no significant differences in nociceptive processing in both groups. Contrary to earlier findings from animal studies, we did not find an effect of metoprolol on the thalamus in either group. However, using a more liberal and exploratory threshold, hypothalamic activity was slightly increased under metoprolol in patients and migraineurs.

**Conclusions:**

No significant effect of metoprolol on trigeminal pain processing was observed, suggesting a peripheral effect of metoprolol. Exploratory analyses revealed slightly enhanced hypothalamic activity under metoprolol in both groups. Given the emerging role of the hypothalamus in migraine attack generation, these data need further examination.

## Background

Beta-blockers such as metoprolol and propranolol are first choice migraine preventive medication. While the clinical efficacy of beta-blockers in reducing migraine attack frequency is certainly established [1–4], it is still poorly understood how they exert their therapeutic effect. So far, no imaging studies investigated the central effects of beta-blockers and our knowledge about the mechanisms derives from preclinical studies. Metoprolol belongs to the group of β-adrenergic blockers and selectively blocks β1 receptors. Beta-blockers attenuate the effects of adrenaline and noradrenaline [5, 6] and thereby downregulate the stimulating effect of the sympathetic nervous system. This downregulation was examined in several measures of cortical information processing that have been shown to be abnormal in migraineurs, such as visual evoked potentials, auditory evoked potentials and contingent negative variations [7]. Beta-blockers seem to have a regulatory effect upon all of these. In the visual system of migraineurs, metoprolol decreased the amplitude of visual evoked potentials [8]. Another study found a decrease of intensity dependence of auditory evoked cortical potentials in migraineurs [9] and this decrease was related to clinical improvement. It has therefore been proposed that modulating the excitability of the cortex is how beta-blockers reduce the migraine attack frequency. Another neurophysiological approach to observe cortical information processing is the analysis of contingent negative variation (CNV), an event-related, slow cerebral potential following activation in the striato-thalamo-cortical loop. In untreated migraineurs the CNV is significantly increased and lacks habituation. Several studies found that beta-blockers normalize the CNV [10] and further that normalization of high CNV was positively correlated with treatment response [10–12]. These studies suggest that the effects refer to a general effect of beta-blockers on cortical excitability and abnormal cortical information processing in migraine. Accordingly it has been hypothesized that beta-blockers exert their preventive action in migraine by modulating cortical excitability and processing [11]. However, the aforementioned studies applied methods with a focus on specific network activity rather than a focus on the location where metoprolol potentially exerts its action in the brain. A plausible explanation for the described abnormalities in sensory processing in migraineurs is a dysfunction of processing in thalamocortical neurons [13–15]. Evidence that preventive action of beta-blockers is effective through β_1_-adrenoceptor inhibition in nociceptive neurons in the thalamus comes from electrophysiological animal studies. Shields and Goadsby (2005) reported that thalamocortical activity evoked by superior sagittal sinus stimulation was inhibited after locally applied propranolol [16].

To address this issue and to achieve a more integrated picture of central effects of metoprolol, we employed pharmacological functional magnetic resonance imaging in combination with a human model of headache attacks. The aim of this study is to assess the effects of metoprolol on brain activation patterns during trigeminal pain in migraine patients, as well as healthy human subjects, determined by fMRI. Based on earlier studies we hypothesize that metoprolol has an inhibiting effect on trigeminal pain processing, especially in the thalamus and/or thalamocortical networks.

## Methods

### Subjects

#### Patients

Twenty five migraineurs were recruited from the Headache Outpatient Department of the University Medical Center Eppendorf, Hamburg. Four patients dropped out after the first session, 2 were excluded because of corrupted data. The final sample therefore encompassed 19 patients (18 females; mean age: 35 ± 2.2 years). Patients fulfilled International Classification of Headache Disorders, 3rd Edition (beta version) criteria of episodic respectively chronic migraine with or without aura [17]. Another inclusion criteria was indication for a preventive medication (more than 3 attacks/months) and the decision to start a treatment with metoprolol. Exclusion criteria were any other neurologic or internal disease, the use of other medications and any contraindication for the MRI examination such as claustrophobia or pregnancy. Clinical characteristics of the patients’ population are included in Table 1.

**Table 1:** Clinical characteristics of the patients population (*n* = 19)

	Episodic migraineurs	Chronic migraineurs	Healthy subjects
Male / female, n	1 / 12	0 / 6	12 / 14
Mean age, y	35	34	25
Patients with aura, n	4	1	N/A
Headaches on scan day I, n	4	2	N/A
Headaches on scan day II, n	3	0	N/A
Headache frequency scan 1, mean d/m	12	19	N/A
Headache frequency scan 2, mean d/m	6	8	N/A
Successful treatment (50% reduction), n	5	4	N/A
Disease duration, mean y	19	16	N/A

#### Healthy subjects

Thrity one healthy subjects were recruited via online advertisements. Exclusion criteria were the presence of any pain disorder (including migraine), neurological and psychiatric disorders as well as any contraindications for metoprolol or the MRI examination. Data of 4 subjects had to be excluded from the analysis for corrupted data and 1 subject dropped out after the first session. Twenty six subjects (14 females; mean age: 25 ± 0.7 years) were included in the final analyses.

#### Standard protocol approvals, registrations, and patient consents

The study was approved by the local ethics committee (PV4084, PV4102) and all subjects gave written informed consent. Subjects were remunerated for participation.

### Study design

#### Healthy subjects

**Fig. 1:**
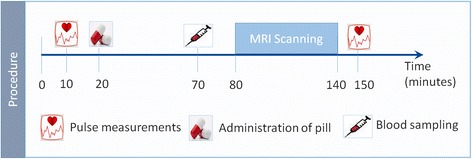
Procedure in healthy subjects group. Timeline of procedures taking place at both scanning sessions (metoprolol and placebo session). Each session started out with pulse measurement and administration of a pill, either treatment or a placebo in a blinded fashion. A blood sample was drawn after a waiting period of 50 min that allowed the drug to reach its maximum plasma concentration while the participant completed the experiment in the scanner. Blood samples were drawn to determine plasma concentration of metoprolol. Then a paradigm of nociceptive trigeminal stimulation was conducted during fMRI, followed by a second pulse measurement after completion

Every subject participated in two identical sessions that were separated by at least 2 weeks to account for the wash-out effect. In a placebo-controlled, crossover, randomized and double-blind fashion, participants took an oral dose of either 75 mg metoprolol or placebo. After a 50 min waiting period to reach the peak plasma concentration of metoprolol [18, 19] during the MR measurement, blood samples were drawn. Procedure of the healthy subjects group is depicted in Fig. 1.

#### Patients

Patients also participated in two identical sessions. One session took place before they started the preventive treatment with metoprolol, the second session when they had taken metoprolol on a regular basis for at least 2 months.

#### Experimental paradigm

**Fig. 2:**
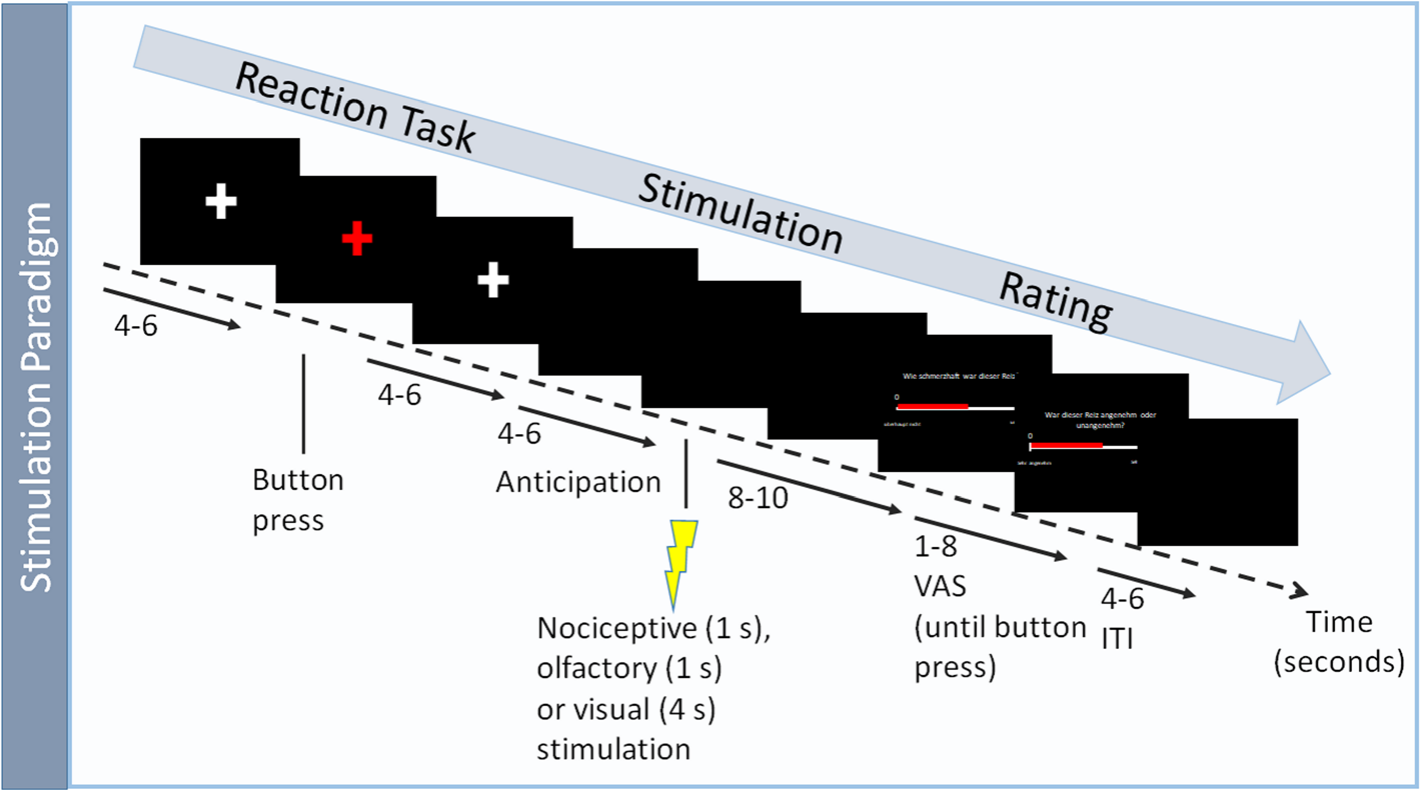
Single trial of stimulation paradigm. Each trial started with a short reaction task, followed by an anticipation phase of jittered length and stimulation with one of the 4 stimuli: 3 gaseous (ammonia, rose odour and air) or a visual stimulus (rotating checkerboard). At the end of each trial subjects rated stimulus painfulness/intensity and pleasantness on two visual analogue scales (VAS). Abbreviations: ITI, Inter-trial interval

The paradigm was identical for healthy subjects and patients in both sessions. In their first session subjects completed a training session to get acquainted with the task before starting the MR session. The standardized trigeminal nociceptive stimulation in the MR scanner (Fig. 2) has been described in detail in previous publications [20, 21]. In summary the paradigm consisted of 4 stimuli: 3 gaseous stimuli and one visual stimulus. Three gaseous stimuli were either ammonia as a nociceptive trigeminal stimulus, rose odor as an olfactory stimulus and air as a control stimulus, applied through a Teflon tube to the left nostril. A rotating checkerboard as a visual stimulus and the gaseous stimuli were applied 15 times each, in a pseudorandomized order. Each stimulation was followed by two visual rating scales, where the subject rated the stimulus painfulness (following the ammonia stimuli) and unpleasantness or intensity (rose and air stimuli) and unpleasantness. Painfulness/intensity was rated on a visual numeric analogue scale from 0 to 100, whereas for unpleasantness rating a bipolar scale from −50 (no sensation) to 50 (very unpleasant) was used. The stimulation paradigm is illustrated in Fig. 2.

### Medication (Metoprolol)

Participants received either metoprolol (75 mg; Metoprolol-ratiopharm®; ratiopharm GmbH, Ulm, Germany) or placebo (Mannitol 99.5 T, highly-dispersed silicon dioxide 0.5 T) in the first session and the alternate substance in the second session. In between measurements a 2-week washout period took place. 50 min after administration blood samples were drawn to determine metoprolol plasma concentration. Blood pressure and heart rate were monitored during the whole session.

### Plasma concentration

At each session a blood sample was drawn from the forearm of the subject using a 4.9 ml vacuum tube containing ethylenediaminetetraacetic acid (EDTA). Afterwards the blood was centrifuged at 2.0 rmp for 20 min at 4 °C and stored at −20 °C until analysis. The Institute of Experimental Pharmacology and Toxicology (Center of Experimental Medicine, University Medical Center Hamburg-Eppendorf, Germany) conducted the analysis of blood plasma concentration via liquid chromatography/mass spectrometry [22].

### MRI data acquisition

All magnet-resonance imaging was acquired on a Siemens Trio 3 T scanner (Siemens AG, Erlangen, Germany) using a 32-channel head coil. High resolution T1 weighted structural images (voxel size 1 m^3^) were obtained using a magnetization-prepared rapid gradient echo sequence. After the structural image, functional images were acquired by an echo planar imaging sequence (repetition time 2.62 s, echo time 30 milliseconds, flip angle 80°, field of view 220 × 220 mm). Each volume consisted of 40 axial slices (slice thickness 2 mm, gap 1 mm). For the whole experiment, scantime was about 55 min.

### Behavioral data analysis

Behavioral data analyses were performed using SPSS Statistics version 22.00 (IBM Corp., Armonk, NY). Ratings were assessed with a visual analogue rating scale (VAS) and mean pain ratings (following trigemino-nociceptive stimulation) were calculated per subject, per session (treatment, no treatment). A paired t-tested was applied in order to compare average pain intensity ratings between sessions at a statistical threshold of *p* < 0.05.

### MRI data processing

Data processing was performed similarly in the healthy subjects and the patients group.

#### Preprocessing

The Statistical Parametric Mapping software SPM12 (Wellcome Trust Centre of Neuroimaging, London, UK) was used for data processing. Standard algorithms and parameters were used, unless specified differently. The first 5 volumes of each session were discarded to account for T1 saturation effects. Anatomical images of each subject were co-registered with the corresponding functional images. The functional images were slice time corrected and realigned to the mean functional image, then normalized into MNI (Montreal Neurological Institute) space and finally smoothed using a 6 mm^3^ Gaussian kernel.

#### Single subject analysis

The general linear model on single subject level included 22 regressors, 11 per session. For each session, experimental regressors included all 4 conditions (ammonia, rose odor, air puffs and visual rotating checker board) as well as button presses. At event onset, these were modeled by convolving stick functions with the canonical hemodynamic response function. Additionally, 6 movement regressors of no interest, resulting from the realignment step, were included per session. The contrast ammonia > air was defined as contrast of interest and also compared between medication and placebo on single subject level.

#### Group analysis

For group analyses the single subject contrast images were entered into the second level. In each group (patients and healthy subjects), potential changes in nociceptive processing caused my metoprolol were assessed by a one-sample t-test that compared BOLD signals (during painful stimulation) between both sessions. Results are reported at a voxel-wise FWE-corrected threshold of *p* < 0.05. Following the a-priori hypothesis that metoprolol acts on the thalamus [16] a small volume correction (SVC: p_(FWE)_ < 0.05) was performed. A thalamus mask obtained from the Harvard-Oxford cortical/subcortical structural atlas (http://www.cma.mgh.harvard.edu/fsl_atlas.html) was used for this analysis. Furthermore we were interested if there are any regions activated under metorpolol during pain in patients and healthy subjects alike. For this exploratory analyses a liberal threshold of 0.005 uncorrected was applied.

## Results

### Behavior

Ammonia stimuli, measured by the VAS scale (±SEM), were rated as painful in the healthy subjects group with a mean of 63.3 (± 2.9) in the metoprolol and 66.7 (±2.4) in the placebo session. In the patients group the mean intensity in the treatment session was 72.5 (± 2.7) and 72.2 (± 2.6) before treatment. Behavioral analysis of intensity ratings did not yield any significant results, neither in the patients, nor in the healthy subjects group.

### Physiology in healthy subjects group

**Fig. 3:**
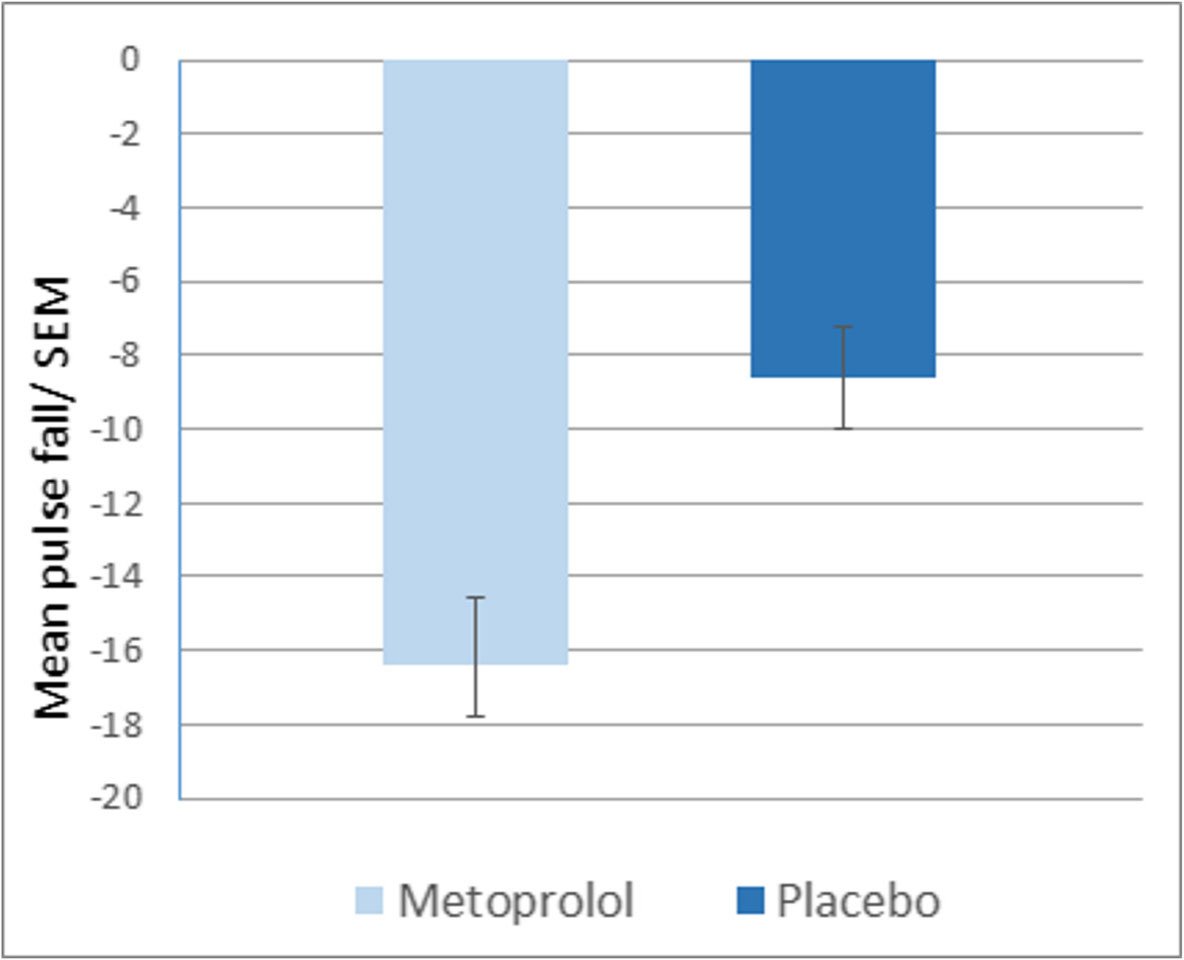
Pulse under metoprolol and placebo in healthy subjects. Pulse of the subjects were measured at two time points in each session and subtracted afterwards (pulse change = T2 [after experimental paradigm] - T1 [before treatment]). The pulse drop in the metoprolol session is significantly bigger than in the placebo session (*p* < 0.001)

The mean metoprolol plasma concentration in healthy subjects, measured in the metoprolol session was 191 ng/mL (SD = 0.8). Additionally we compared the mean drop of heart rate (beats/min) in both sessions (Fig. 3). In each session, we subtracted the heart rate measured after the experiment, from the heart rate measured before the medication/placebo pill was administered (heart rate T2 – heart rate T1). In the metoprolol session the mean drop of the heart rate was 16.4 (±1.8), whereas in the placebo session it was 8.6 (±1.4). The fall of the heart rate in the metoprolol session was significantly larger than in the placebo session (*p* < 0.001).

### Imaging

#### Main effects of painful trigeminal stimulation

In both groups, we detected significantly increased neural activation (*p* < 0.05, voxel-wise FWE-corrected) during nociceptive trigeminal stimulation in several pain related cortical and subcortical areas. The increase in BOLD signal responses included the bilateral thalamus, insular cortex, midcingulate and anterior cingulate cortex (MCC, ACC), cerebellum as well as somatosensory cortices and brainstem areas.

#### Differences in pain processing between metoprolol and placebo

We found no difference between the metoprolol and placebo session in BOLD signal intensity (*p* < 0.05, voxel-wise FWE-corrected) during trigeminal pain. Contrary to earlier studies, we did not find any inhibition in BOLD signal intensity of the thalamus after metoprolol treatment compared to placebo (SVC). The opposite contrast did not reveal any differences either.

#### Exploratory analyses

**Fig. 4:**
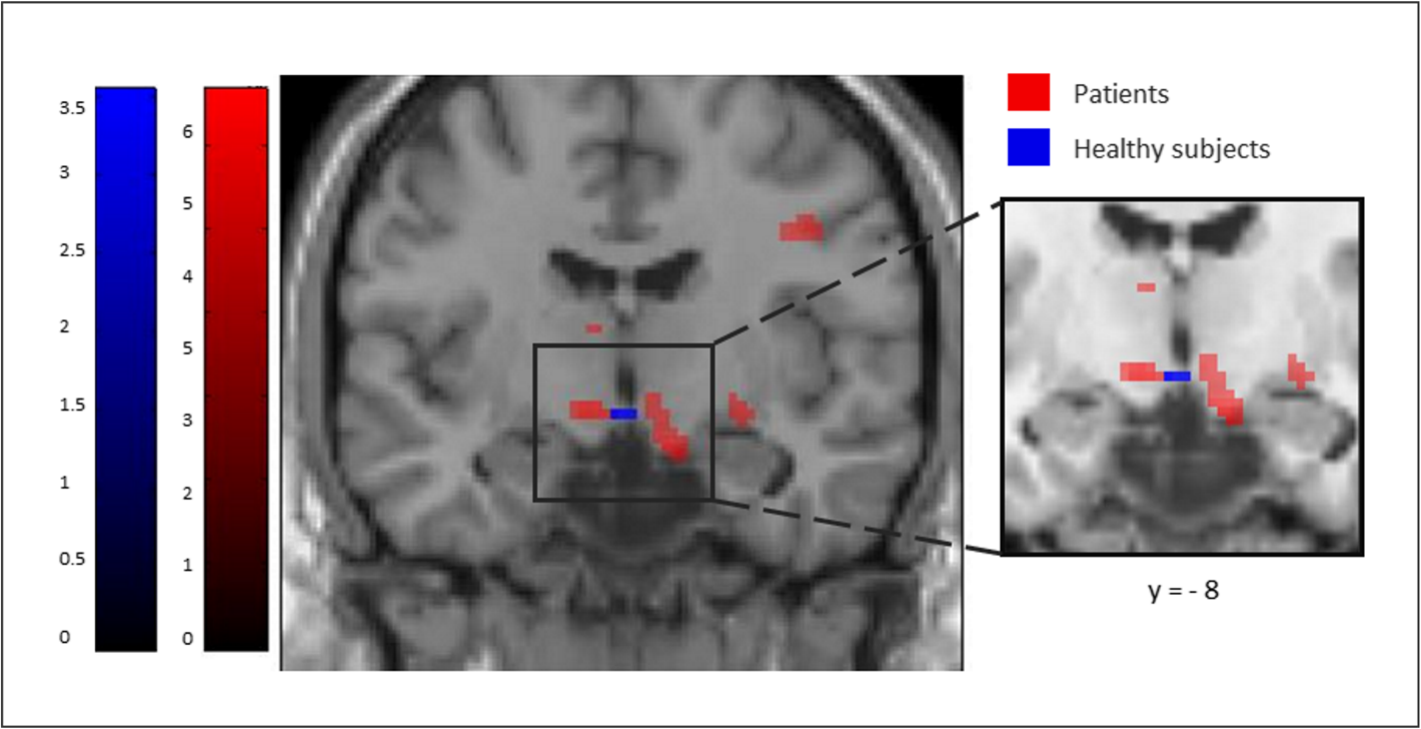
Increased hypothalamic activation under Metoprolol during pain. At an exploratory threshold of *p* < 0.005 (uncorrected), hypothalamic activity is similarly increased in patients (red) and healthy subjects (blue) under metoprolol during trigeminal stimulation

**Fig. 5:**
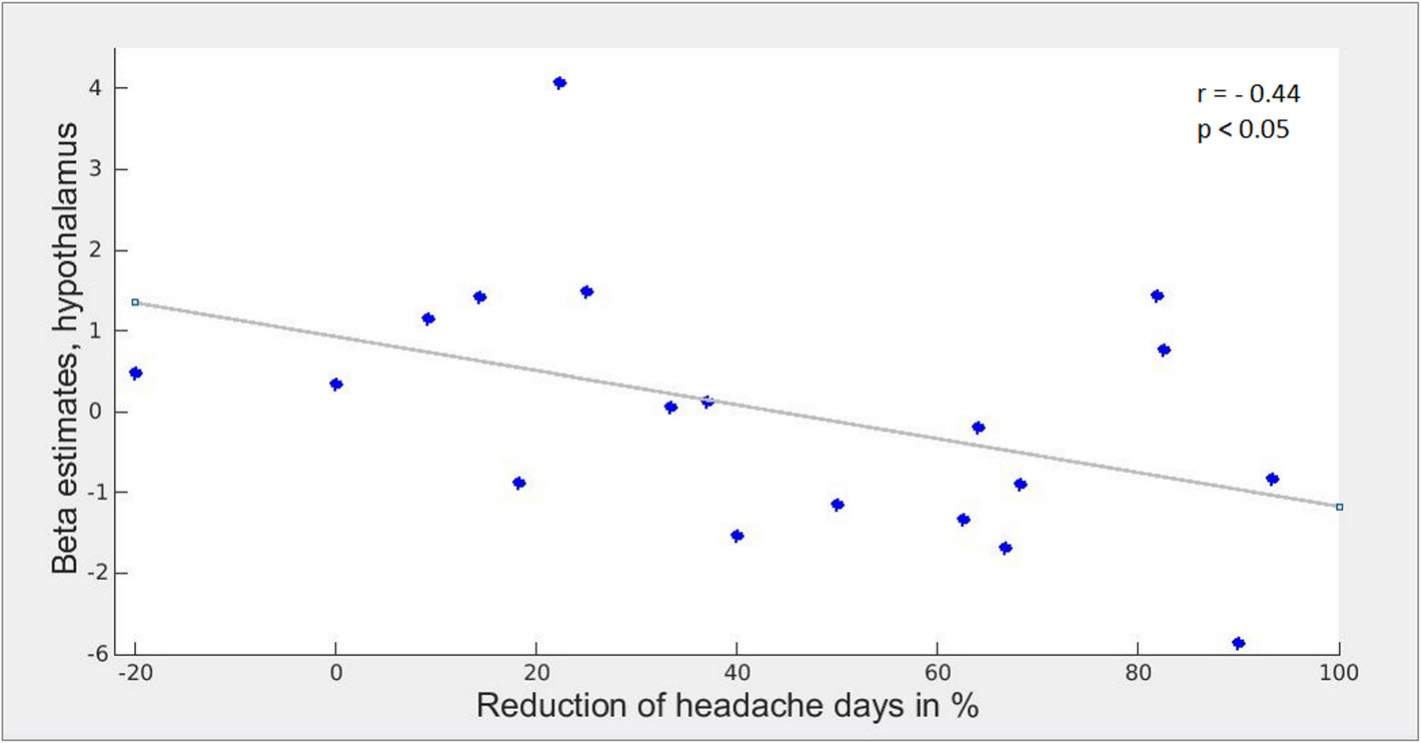
Relationship of hypothalamic activity and the reduction of headache days in patients under metoprolol treatment. A bigger reduction of headache days in patients, marked by a higher percentage of reduction, is related to lower beta estimates within a cluster in the left hypothalamus (peak: x = −8, y = −8, z = −10)

To further explore the effect of metoprolol on trigeminal pain processing with regard to similarities under metoprolol (during nociceptive input) in both groups, patients and healthy subjects, we lowered the threshold to 0.005 uncorrected. In both groups, i.e. patients and healthy subjects, we found the BOLD signal intensity of the hypothalamus increased under metoprolol during pain, compared to no medication, placebo respectively (Fig. 4). Following these results, we had a closer look at the relationship of hypothalamic activity (MNI coordinates (peak) and size of the significant cluster: x = −8, y = −8, z = 9, k = −10), in patients with the treatment effect of metoprolol. As depicted in Fig. 5, we found a negative correlation (*r* = −0.44, *p* < 0.05) between betavalues in the hypothalamus and a reduction of headache days. The bigger the reduction of headache, the fewer hypothalamic activity.

## Discussion

Contrary to our hypotheses, we found no significant effect of metoprolol on central trigeminal pain processing, neither in patients, nor in healthy subjects. The healthy subject group showed metoprolol plasma concentrations and a significant drop of heart rate under metoprolol compared to placebo, demonstrating a biological effect of the single dosage. Either, this effect on the vegetative nervous system is not reflected in central neural structures or, more likely, it is due to peripheral activity only. In either case, the results do not support the notion that beta-blockers act centrally, as suggested by experimental animal studies [16]. However, a purely peripheral site of action of beta-blockers ignores their effect on attack frequency, premonitory symptoms and side effects of central nervous origin [23, 24]. One could argue that the administered dose of 75 mg might have been too low to reach a neural level after a single dose, but this is unlikely given the above mentioned biological effect of the single dosage that we observed. We chose this dosage as this was ethically justifiable and 75 mg per day is the usual dose for migraine prevention. Nonetheless, metoprolol may indeed only exert its central effects leading to a reduction in attack frequency, if taken on a regular basis over several weeks. This was for ethical reasons not feasible in healthy volunteers, for which reason we have also investigated migraine patients who took metoprolol daily over several months before treatment and under treatment. But also in the group of migraineurs, no significant effect of metoprolol on central pain processing was observed. Another possible explanation for the missing effect of metoprolol is that the functional changes caused by metoprolol may be too subtle to be disclosed by functional MRI.Exploring the data further, we used a more lenient threshold of 0.005 (uncorrected) and interestingly found the hypothalamus being more activated following nociceptive input during metoprolol treatment in both groups, patients and healthy subjects. This suggests that beta-blockers may modulate hypothalamic action and that this modulation has an essential role in its preventive effect. Given the hypothalamus’ role in the pathophysiology of migraine pain [25] and chronification [26], it would be a conceivable target for preventive migraine medication such as metoprolol. Following this line of investigation, we correlated hypothalamic activity in patients with drug effectiveness and found a negative correlation between hypothalamic activity and reduction of headache days, i.e. the stronger the reduction of headache days, the fewer hypothalamic activity. These speculations have to be seen with caution, as changes in hypothalamic activity were detected only at an exploratory threshold. Nevertheless it is an interesting finding and encourages further investigation of the hypothalamus as a possible target of metoprolol in preventive treatment. An interesting fact of this study is that the hypothalamus already increased after just a single treatment of metoprolol in healthy subjects. It would be interesting to see whether a longer treatment phase would affect physiological phenomenon or (stress) thresholds of healthy participants. However, given that it is still not clear whether central effects of metoprolol determine its therapeutic effect in migraine or if its therapeutic effect is caused by changes in the periphery, the issue merits further studies.

## Conclusion

For the first time, the preventive mechanism of metoprolol in migraine treatment is being investigated with the method of pharmacological imaging, which has successfully been applied to enlighten the pharmacodynamics mechanisms of other medications [27–29]. Taken together, our study did not find an effect of systemically administered metoprolol on central pain processing structures, including the thalamus, neither in the healthy system, nor in the pathological system of migraineurs.

## Abbreviations

ACC: Anterior cingulate cortex
BOLD: Blood Oxygen Level Dependent;
fMRI: Functional Magnetic Resonance Imaging
FWE: Familywise Error
i.e.: id est.
ITI: Inter-trial interval
MCC: Midcingulate cortex
MR: Magnetic Resonance
SVC: Small volume correction
VAS: Visual analogue scale
